# Prevalence and Correlates of Environmental Enteric Dysfunction Among Young Children in Bangladesh

**DOI:** 10.1111/mcn.70237

**Published:** 2026-09-01

**Authors:** Lauren Thompson, Charles D. Arnold, Julie M. Long, Jamie L. E. Westcott, Rahvia Alam Sthity, Afsana Mim Khandaker, M. Munirul Islam, Robert E. Black, Nancy F. Krebs, Christine M. McDonald

**Affiliations:** ^1^ Institute for Global Nutrition University of California Davis California USA; ^2^ Department of Pediatrics, Section of Nutrition University of Colorado Anschutz Medical Campus Aurora Colorado USA; ^3^ International Centre for Diarrhoeal Disease Research Bangladesh (icddr,b) Dhaka Bangladesh; ^4^ Bloomberg School of Public Health Johns Hopkins University Baltimore Maryland USA; ^5^ International Zinc Nutrition Consultative Group San Francisco California USA; ^6^ Department of Pediatrics, Division of Gastroenterology, Hepatology and Nutrition University of California San Francisco California USA

## Abstract

Bangladesh continues to experience high levels of child undernutrition, with stunting affecting approximately 24% of children under 5 years of age. Environmental enteric dysfunction (EED), a subclinical intestinal disorder characterized by chronic inflammation, immune activation, and nutrient malabsorption, may reduce the effectiveness of nutritional interventions. Identifying factors that may enhance resilience to EED could guide intervention design. This study measured two biomarkers of EED, fecal calprotectin (CAL) and myeloperoxidase (MPO), among 160 Bangladeshi children 9–11 months of age and assessed associations between these biomarkers and indicators of child growth, micronutrient status, and inflammation using multivariate logistic regression. Outcomes were defined as CAL, MPO, or both biomarkers below the 25th percentile. CAL (> 160 µg/g) and MPO (> 2000 ng/mL) were elevated in over 90% and 70% of children, respectively, indicating widespread intestinal inflammation. In multivariable models, higher mid‐upper arm circumference‐for‐age Z‐scores (MUAC‐Z) were associated with lower CAL (OR 1.51, 95% CI 1.04–2.18) and combined low CAL and MPO values (OR 1.72, 95% CI 1.06–2.81). Higher retinol‐binding protein (RBP) was strongly associated with lower MPO (OR 4.91, 95% CI 1.28–18.83), while lower α‐1‐acid glycoprotein (AGP) levels were associated with lower MPO values (OR 0.53, 95% CI 0.27–1.04). Better ponderal growth, adequate vitamin A status, and lower systemic inflammation characterized children with lower concentrations of EED biomarkers. Reducing the burden of EED may help to enhance the effectiveness of nutrition interventions.

## Summary

- Environmental enteric dysfunction (EED) is a subclinical condition characterized by chronic intestinal inflammation, structural mucosal alterations and nutrient malabsorption, and is prevalent among young children in many lower‐ and middle‐income countries.
- This study leveraged micronutrient and inflammatory biomarkers, as well as anthropometric measurements, to explore associations with fecal calprotectin (CAL) and myeloperoxidase (MPO), biomarkers of EED, among children 9‐11 months old in Dhaka, Bangladesh.
- CAL and MPO were elevated in over 90% and 70% of children, respectively, indicating widespread intestinal inflammation consistent with EED.
- Our findings suggest that reducing the burden of EED may help to improve the effectiveness of nutrition interventions. These findings are limited by the percentile‐based reference group, cross‐sectional design, and reliance on only two EED biomarkers (CAL and MPO).

## 1 Introduction

Undernutrition is responsible for approximately half of all child mortality globally (United Nations Children's Fund UNICEF, W.H.O. 2023). Bangladesh continues to experience a high burden of child undernutrition, with stunting affecting approximately 24% of children under 5 years of age (National Institute of Population Research and Training NIPORT and ICF 2024). Micronutrient deficiencies are also widely prevalent; zinc deficiency, vitamin A deficiency and iron deficiency affects approximately 31%, 51% and 21% of preschool‐aged children respectively, according to the most recent National Micronutrient Survey in Bangladesh (National Micronutrient Survey in Bangladesh 2019–2020). Undernutrition can bear significant long‐term health consequences for children, including heightened vulnerability to infections and significant deficits in cognitive and physical development (De Sanctis 2021; Victora 2021).

A fundamental determinant of a child's nutritional status is intestinal health and function, given its central role in nutrient absorption and utilization (Farré 2020). Environmental enteric dysfunction (EED), a subclinical disorder characterized by chronic intestinal inflammation and structural alterations, significantly impairs intestinal health (Syed et al. 2016). This condition arises primarily from chronic fecal‐oral contamination by enteropathogens and is especially prevalent in resource‐limited settings with inadequate sanitation infrastructure (Tickell et al. 2019a). Biomarker and biopsy data suggest that EED is highly prevalent among young children in several different lower‐middle income country settings, including Bangladesh (Kortekangas 2022; Hossain 2023; Lin 2020; Long 2019; Fahim 2018; Iqbal 2018; Harper 2018; Kosek 2017). Specifically, a recent study in Dhaka identified that over 90% of children 12–18 months old displayed intestinal damage consistent with EED (Hossain 2023).

Evidence suggests that morphological changes induced by EED in the small intestine result in nutrient malabsorption and compromised intestinal barrier function (Tickell et al. 2019b). This allows the translocation of luminal contents, including pathogenic bacteria, into systemic circulation, provoking systemic inflammation (Hossain 2023; Tickell et al. 2019b; Trehan 2016; Keusch 2014). This inflammatory response may further exacerbate nutritional deficiencies and growth faltering (Syed et al. 2016; Tickell et al. 2019b; Harper 2018; Owino 2016), creating a detrimental feedback loop that could diminish the effectiveness of nutritional interventions. Therefore, elucidating the relationships between key biological factors and EED may give clues to the underlying mechanisms of EED. An improved understanding of these mechanisms may inform intervention strategies aimed at reducing EED and enhancing the effectiveness of nutritional interventions among vulnerable child populations.

The objective of the present study is to measure two biomarkers of EED, fecal calprotectin (CAL) and myeloperoxidase (MPO), among a subpopulation of young Bangladeshi children between 9 and 11 months of age enrolled in the Zinc in Powders Trial (ZiPT), which took place in Dhaka, Bangladesh. We also aim to identify anthropometric indicators and biomarkers of micronutrient status and inflammation that are associated with CAL, MPO, or both biomarkers below the 25th percentile in our study population.

## 2 Methods

### 2.1 Study Design, Setting and Population

The present study is a sub‐study of the ZiPT (Islam 2022): a randomized, partially double‐blind, controlled, community‐based efficacy trial that was conducted in the peri‐urban, low‐income area of Mirpur in Dhaka, Bangladesh. The study site was selected for its proximity to the International Center for Diarrheal Disease Research, Bangladesh (icddr,b) and its high prevalences of zinc deficiency, diarrhea and stunting among young children under 5 years of age. The study protocol for the main trial has been published (Islam 2018).

A total of 174 children 9–11 months of age were enrolled in the sub‐study. Children in this study also participated in the biochemical subgroup of the ZiPT parent trial, which was restricted to children randomized to the daily 10 mg zinc tablet, daily micronutrient powder (MNP) containing 10 mg zinc and 6 mg iron, and placebo arms (Long 2022). Only baseline samples from these participants were included in the present analysis. Children were eligible for enrollment if they were 9–11 months of age and had a weight‐for‐length z‐score (WLZ) ≥ −3 according to the 2006 WHO Growth Standards. Children were excluded from the trial if they had any of the following conditions: (1) severe acute malnutrition, defined as WLZ < − 3 and/or the presence of bipedal edema and/or mid‐upper arm circumference (MUAC) < 115 mm; (2) severe anemia (hemoglobin concentration < 8 g/dL, as measured by single‐drop capillary blood collected via fingerprick using Hemocue 301+); (3) congenital anomalies (e.g., cardiac defects, cleft lip or palate) or any other conditions that interfere with feeding; and (4) chromosomal anomalies and other organic problems (e.g., jaundice, tuberculosis, etc.). Enrollment took place between September 2018 and July 2019.

### 2.2 Data Collection

At enrollment, self‐reported data by the participant's mother on background, socioeconomic status, maternal education level, household characteristics, sanitation and handwashing practices, source of drinking water, ownership of assets, and food security were collected using pretested questionnaires. Certain variables were used to estimate composite household asset scores and hygiene scores. Asset score was defined using the sum of 2 subscores: asset score 1, which was the sum of the following possessions: iron, chair/bench, sofa, table, computer, fridge, motorcycle, and bank account; asset score 2, which was the sum of the following possessions: electric fan, television, mattress, and mobile phone. Asset score ranged from 0 to 12. Hygiene score was calculated as the sum of the scores of the following variables: “Wash hands after helping child defecate”; “Wash hands before preparing food,” “Wash hands after using toilet”; and “Uses toilet paper”. Each variable was scored as follows: 1 = never, 2 = rarely, 3 = sometimes, 4 = always. Asset score could range from 4 to 16. Household food security was assessed using the Household Food Insecurity Access Scale (Coates et al. 2007).

Body weight and length of each child were measured by trained study anthropometrists. Body weight was measured on a balance sensitive to 2 g (SECA, model No. 7281321009, Hamburg, Germany) and length was measured to 0.1 cm using an infantometer (SECA, model No. 4161721009, Hamburg, Germany). The measurements were performed in triplicate following standard procedures (De Onis 2004). Stunting, underweight and wasting were defined as length‐for‐age z‐scores, weight‐for‐age z‐scores and WLZ < ‐2 SD, respectively (WHO Multicenter Growth Reference Study Group, 2006).

Blood samples were collected at the Clinical Trials Unit on the icddr,b campus in Dhaka. A trained phlebotomist collected 5 mL of venous whole blood from the antecubital vein of participants. Blood samples were drawn in the morning hours when the participants were fasted and were collected into certified trace element‐free tubes (Sarstedt, Numbrecht, Germany) using aseptic techniques and IZiNCG recommendations for the avoidance of contamination (International Zinc Nutrition Consultative Group 2019). Blood samples were immediately refrigerated and centrifuged for separation of serum. Serum samples were then aliquoted into two tubes: (1) a trace element‐free cryovial for analysis of serum zinc concentrations (SZC), and (2) a Sarstedt cryovial for analysis of ferritin, soluble transferrin receptor (sTfR), retinol‐binding protein (RBP), alpha‐1‐glycoproetin (AGP), and C‐reactive protein (CRP). These aliquots were stored at −20°C and shipped to the Pediatric Nutrition Laboratory, University of Colorado, and the VitMin Laboratory (Willstaet, Germany) for laboratory analysis. Lastly, hemoglobin concentrations were assessed using a Hemocue 301+ (Hemocue; Ångelholm, Sweden) using capillary blood samples collected via fingerprick. Micronutrient deficiencies were defined according to the following cutoffs: (1) zinc deficiency: SZC < 65 µg/dL; (2) anemia: hemoglobin < 10.5 g/dL; (3) iron deficiency: inflammation‐adjusted ferritin < 12 g/L, (4) iron deficiency anemia: inflammation‐adjusted ferritin < 12 ug/L and hemoglobin < 10.5 g/dL; (5) vitamin A deficiency: inflammation‐adjusted retinol‐binding protein (RBP) < 0.81 µmol/L (Baeten 2004). Inflammation was defined as CRP > 5 mg/L or AGP > 1 g/L.

Stool samples were collected from children in sterile collection tubes with the assistance of a health worker. An aliquot of the stool sample was transferred to the Emerging Infections and Parasitology Laboratory at icddrb, maintaining a cold chain. Aliquots were stored at −70°C or colder until further analysis. Fecal CAL was quantified using a Calprotectin ELISA kit (Cat. #EK‐Cal‐2, BÜHLMANN Laboratories AG, Basel, Switzerland) following the manufacturer's protocol. A standard curve was constructed from the standard wells, applying a 4‐parameter logistic curve for fit. The optical density of each sample was measured at 450 nm wavelength using an ELISA reader (BioTek, Agilent, CA, USA). Calprotectin concentrations were determined from the standard curve and the results expressed in µg/g. Fecal MPO levels were measured using a Myeloperoxidase ELISA kit (Cat. #KR6630, Immundiagnostik AG, Bensheim, Germany), following the manufacturer's protocol. A standard curve was constructed, and absorbance was measured at 450 nm wavelength using the same ELISA reader. MPO concentrations were determined from the standard curve, adjusted by a final dilution factor of 500, and reported as ng/mL. To estimate the precision of the ELISA assays, known controls were used to assess intra‐ and inter‐assay variation. Samples with concentrations outside the measurement range were further diluted or concentrated and re‐assayed as needed. Elevated CAL was defined as CAL > 160 ug/g, and elevated MPO was defined as MPO > 2000 ng/mL in accordance with the manufacturer's protocols for the respective ELISA kits.

### 2.3 Statistical Analysis

Because a majority of children demonstrated elevated CAL and MPO concentrations, for this analysis we were interested in identifying correlates of CAL and/or MPO concentrations that were in the lowest quartile of our sample population. In this way, we employed a positive deviance approach, in which we aimed to identify potentially protective factors that were associated with lower EED biomarker concentrations among children compared to their peers within the same context who share similar environmental resources and constraints. We defined three primary outcome variables based on biomarker levels: (1) CAL below the 25th percentile, (2) MPO below the 25th percentile, and (3) both CAL and MPO below the 25th percentile. Assuming an outcome prevalence of 0.25, a two‐sided *α* = 0.05, and 80% power, a sample size of 174 children was determined to be sufficient to detect an effect size of 0.49 standard deviations (SDs) difference in continuous correlates between children in the lowest quartile and those in the upper three quartiles.

Descriptive statistics were tabulated for anthropometric measurements, biomarkers of micronutrient status and inflammation, maternal and household characteristics to describe the study population. MPO and CAL were log‐transformed to approximate a normal distribution. Correlation between MPO and CAL values was assessed using Pearson's correlation coefficient. Logistic regression models were used to identify anthropometric variables and biomarkers of micronutrient and inflammatory status that were associated with the primary outcomes in minimally adjusted models, controlling for age. Multivariable models were subsequently fit, including all predictors that were associated with each outcome in minimally adjusted models at a significance level of *p‐*value < 0.1. Collinearity was assessed using variance inflation factors (VIF), and if predictors were significantly correlated, one was selected for inclusion in the multivariable model to represent the underlying construct. Results are presented as adjusted odds ratios (OR) and 95% confidence intervals (CI). Statistical analyses were performed using R (Version 2023.12.1 + 402).

### 2.4 Ethics Statement

Ethical approval for ZiPT was provided by the Institutional Review Boards of icddr,b, Children's Hospital Oakland Research Institute, and the University of Colorado, Denver. All procedures were conducted in accordance with the Declaration of Helsinki. The research protocol was registered at clinicaltrials.gov as NCT03406793 and previously published (Islam et al. 2018). Informed consent was provided by the child's mother or primary caregiver in Bangla, the national language of Bangladesh.

## 3 Results

Of the 174 participants enrolled in the study, 160 participants successfully provided both a stool sample and a venous blood sample and were included in analyses. Background characteristics of child participants are summarized in Table 1. Mean ± SD child age was 9.8 ± 0.9 months, and the prevalences of stunting, underweight, and wasting were 28.1%, 21.2% and 3.1%, respectively. In addition, 9.4% of children had a MUAC measurement < 12.5 cm. Only 23.8% of children were considered healthy at enrollment (had no morbidity symptoms in the past 2 weeks as reported by the child's caregiver). Micronutrient deficiencies were common: 60.6% of children were anemic (hemoglobin < 10.5 g/dL), 54.7% were iron deficient, and 38.4% had iron‐deficiency anemia. The prevalences of zinc deficiency and vitamin A deficiency were 34.8% and 17.6%, respectively. Mothers of children were 24.7 ± 4.8 years of age and completed 5.9 ± 3.7 years of formal education, on average. 58% of households had drinking water piped directly into the home, and the mean ± SD household hygiene score was 12.7 ± 2.4 out of 16. Mean ± SD asset score was 5.3 ± 2.3 out of 12. 16% of households were classified as food‐insecure.

**Table 1:** Baseline characteristics of Bangladeshi children 9–11 months included in the sub‐study (*n* = 160).

Child characteristics	
Age, months	9.8 (0.9)
Female	79 (49.4%)
Breastfeeding	148 (92.5%)
Length‐for‐age Z‐score	−1.4 (1.1)
Weight‐for‐age Z‐score	−1.2 (1.1)
Weight‐for‐length Z‐score	−0.6 (0.9)
Stunted^a^	45 (28.1%)
Wasted^b^	5 (3.1%)
Underweight^c^	34 (21.2%)
MUAC, cm	13.8 (1.0)
MUAC < 12.5 cm	15 (9.4%)
Child is healthy at enrollment	38 (23.8%)
Hemoglobin (g/dL)	10.2 ± 1.0
Anemic (Hemoglobin < 10.5 g/dL)	97 (60.6%)
Adjusted ferritin^d^ (ug/dL)	10.2 [5.0, 19.8]
Iron deficiency (Adjusted ferritin^d^ < 12 ug/dL)	87 (54.7%)
Iron deficiency anemia (Hb < 10.5 g/dL and adjusted ferritin^d^ < 12 ug/dL)	61 (38.4%)
Serum zinc (ug/dL)	70.3 ± 13.1
Zinc deficiency (Serum zinc < 65 ug/dL)	55 (34.8%)
RBP (umol/L)	1.0 ± 0.3
Vitamin A deficiency (RB*p* < 0.81 umol/L)	28 (17.6%)
Inflammation	
CRP > 5 mg/L	13 (8.2%)
AGP > 1 g/L	35 (22.0%)
Maternal Characteristics	
Age, years	24.8 ± 4.8
Years of education completed	5.9 ± 3.7
Parity	2.0 (1.0)
Socioeconomic Characteristics	
Number of household members	4.6 ± 1.3
Source of drinking water	
Piped into dwelling	7 (4.4%)
Piped into yard/plot	94 (58.8%)
Other	59 (36.9%)
Hygiene score^e^	12.8 ± 2.4
Asset score^f^	5.3 ± 2.3
HFIAS^g^ classification	
Food‐insecure	26 (16.2%)
Food‐secure	134 (83.8%)

*Note:* Values are means ± SD, median [IQR] or *n* (%). Abbreviations: AGP, Alpha‐1‐acid glycoprotein; BMI, body mass index; CRP, C‐reactive protein; MUAC, middle‐upper arm circumference; RBP, retinol‐binding protein.

^a^ Stunted defined as a length‐for‐age z‐score < −2 standard deviations.

^b^ Wasted defined as a weight‐for‐length z‐score < −2 standard deviations.

^c^ Underweight defined as weight‐for‐age z‐score < −2 standard deviations.

^d^ Ferritin concentration was adjusted for CRP and AGP using the BRINDA regression correction approach.

^e^ Hygiene score was calculated as the sum of the scores for the following variables: “Wash hands after helping child defecate”; “Wash hands before preparing food”; “Wash hands after using toilet”; and “Uses toilet paper”. Each variable was scored as follows: 1 = never, 2 = rarely, 3 = sometimes, 4 = always.

^f^ Asset scores were the sum of two sub‐scores: asset score 1, which was the sum of the following possessions: iron, chair/bench, sofa, table, computer, fridge, motorcycle, and bank account; asset score 2, which was the sum of the following possessions: electric fan, television, mattress, mobile phone. Asset score could range from 4 to 16.

^g^ As defined by the Household Food Insecurity Asset Scale (HFIAS).

**Figure 1:**
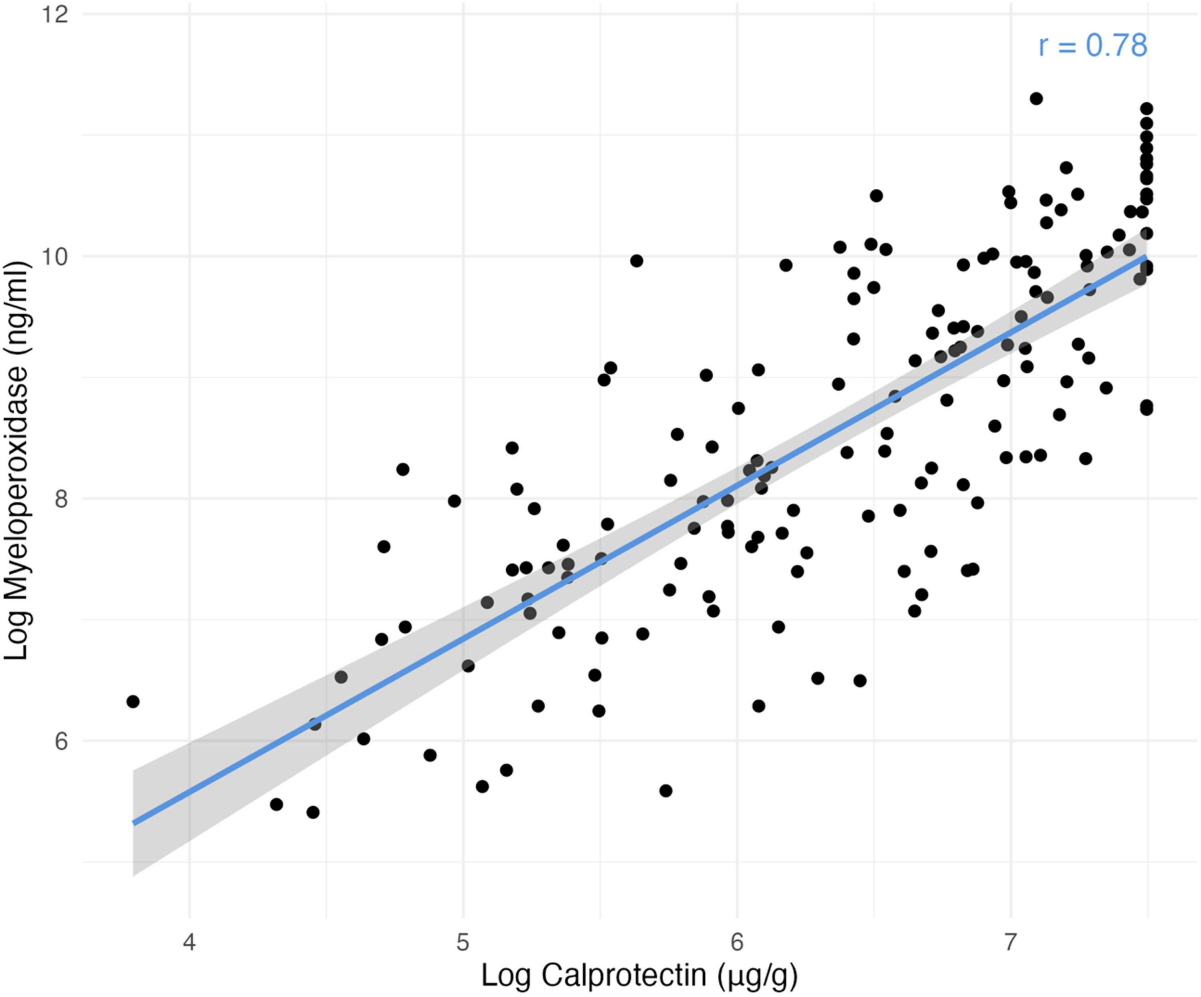
Scatterplot of fecal calprotectin and myeloperoxidase values.

Regarding biomarkers of EED, median [IQR] CAL concentrations were 681.2 ug/g [316.1, 1170.8] and 4812 ng/mL [1,741, 19,168.4], respectively. The prevalences of elevated CAL and elevated MPO were 91.3% and 72.5%, respectively (Table 2). CAL and MPO biomarkers were highly correlated (Figure 1), with a correlation coefficient of *r* = 0.78 (*p‐*value < 0.001). Forty children fell into the lowest quartile of CAL and 40 into the lowest quartile of MPO when considered separately; 32 children overlapped in the lowest quartile for both biomarkers. In minimally‐adjusted logistic regression models, children with a higher MUACZ were more likely to have CAL concentrations in the lowest quartile (OR 1.51, 95% CI 1.04–2.18, *p‐*value = 0.03). WLZ exhibited a similar association (OR 1.45, 95% CI 0.99–2.14, *p‐v*alue = 0.06). Higher RBP (OR 4.91, 95% CI 1.28–18.83, *p‐*value = 0.02) and hemoglobin concentrations (OR 1.35, 95% CI 0.93–1.95, *p‐*value = 0.08) were associated with increased odds of lower MPO. Children with higher AGP concentrations were marginally less likely to have lower MPO concentrations (OR 0.53, 95% CI 0.27–1.04, *p‐*value = 0.07). Children with a higher MUACZ (OR 1.72, 95% CI 1.06–2.81, *p‐*value = 0.03) were more likely to have lower CAL and MPO values. Increased RBP concentrations (OR 3.90, 95% CI 0.90–17.01, *p‐*value = 0.07), higher WAZ (OR 1.48, 95% CI 0.99–2.21, *p‐*value = 0.07) and greater WLZ (OR 1.72, 95% CI 1.11–2.68, *p‐*value = 0.06) were marginally associated with lower CAL and MPO values (Table 3).

**Table 2:** Fecal calprotectin and myeloperoxidase: biomarker concentrations and prevalence of elevated biomarkers among children enrolled in ZiPT.

EED biomarker concentrations	Median [IQR]
Calprotectin (ug/g)	681.2 [316.1, 1,170.8]
Myeloperoxidase (ng/mL)	4812.0 [1741.0, 19,168.4]

Prevalence of elevated biomarkers^a^ of EED	*n* (%)
Calprotectin > 160 ug/g	146 (91.3%)
Myeloperoxidase > 2000 ng/mL	116 (72.5%)

^a^ Elevated biomarker concentrations defined by ELISA kit manufacturer (Calprotectin kits: Bühlmann Labs, Myeloperoxidase kits: Immundiagnostik).

**Table 3:** Predictors of calprotectin < 25th percentile and myeloperoxidase < 25th percentile: minimally adjusted models.

Predictor variable	Outcome					
	Calprotectin < 25th Percentile^a^		Myeloperoxidase < 25th Percentile^a^		Myeloperoxidase & Calprotectin < 25th Percentile^a^	
	OR (95% CI)	*p*‐value	OR (95% CI)	*p*‐value	OR (95% CI)	*p*‐value
Biochemical variables						
Hemoglobin (g/dL)	0.81 (0.55, 1.17)	0.26	1.35 (0.93, 1.95)	0.08	1.02 (0.67, 1.55)	0.94
Adjusted ferritin^b^^,^ d (ug/L)	0.76 (0.52, 1.11)	0.16	0.96 (0.66, 1.40)	0.96	0.77 (0.48, 1.15)	0.18
sTfR^d^ (mg/L)	1.07 (0.46, 249)	0.88	0.76 (0.32, 1.79)	0.26	0.91 (0.35, 2.39)	0.55
Adjusted BIS^c^ (mg/kg body weight)	0.95 (0.87, 1.03)	0.24	1.00 (0.92, 1.09)	0.65	0.95 (0.87, 1.05)	0.32
RBP (umol/L)	1.74 (0.48, 6.33)	0.40	4.91 (1.28, 18.83)	0.02	3.90 (0.90, 17.01)	0.07
Serum zinc (ug/dL)	0.99 (0.96, 1.02)	0.50	1.02 (0.99, 1.05)	0.19	1.00 (0.97, 1.03)	0.89
AGP^d^ g/L	0.73 (0.38, 1.41)	0.35	0.53 (0.27, 1.04)	0.07	0.56 (0.26, 1.20)	0.14
CRP^d^ mg/L	0.89 (0.73, 1.08)	0.23	0.86 (0.71, 1.05)	0.15	0.90 (0.75, 1.13)	0.36
Anthropometric variables						
MUAC, cm	1.51 (1.04, 2.18)	0.03	1.31 (0.91, 1.89)	0.14	1.71 (1.12, 2.60)	0.02
MUAC‐for‐age Z‐score	1.58 (1.03, 2.42)	0.04	1.25 (0.83, 1.90)	0.29	1.72 (1.06, 2.81)	0.03
Length‐for‐age Z‐score	1.03 (0.71, 1.47)	0.87	1.08 (0.76, 1.54)	0.66	1.10 (0.74, 1.65)	0.64
Weight‐for‐age Z‐score	1.28 (0.90, 1.82)	0.18	1.21 (0.86, 1.72)	0.27	1.48 (0.99, 2.21)	0.06
Weight‐for‐length Z‐score	1.45 (0.99, 2.14)	0.06	1.28 (0.88, 1.88)	0.20	1.72 (1.11, 2.68)	0.06

Abbreviations: AGP, alpha‐1‐acid glycoprotein; BIS, body iron stores; BMI, body mass index; CI, confidence interval; CRP, C‐reactive protein; MUAC, middle‐upper arm circumference; OR, odds ratio; RBP, retinol‐binding protein; sTfR, serum transferrin receptor.

^a^ Logistic regression models are minimally adjusted for child age. Models were run separately for calprotectin and myeloperoxidase.

^b^ Ferritin concentration was adjusted for CRP and AGP using the BRINDA regression correction approach.

^c^ Body iron stores = −(log10(sTfR/(ferritin/1000)) −2.8229)/0.1207. In this formula, soluble transferrin receptor and ferritin were adjusted for CRP and AGP using the BRINDA regression correction approach.

^d^ Continous ferritin, sTfR, CRP and AGP values were log‐transformed to approximate a normal distribution.

^e^ RBP concentration was adjusted for CRP and AGP using the BRINDA regression correction approach.

In multivariable models, higher MUACZ was associated with increased odds of lower CAL concentrations (OR 1.51, 95% CI 1.04–2.18, *p‐*value = 0.03). WLZ was excluded from the multivariable model as it was collinear with MUACZ. Higher RBP concentrations were associated with increased odds of lower MPO values (OR 6.23, 95% CI 1.59–26.74, *p‐*value = 0.01) and increased AGP concentrations were associated with reduced odds of lower MPO concentrations (OR 0.40, 95% CI 0.17–0.85, *p‐*value = 0.02). Finally, increased MUACZ was associated with an increased odds of lower CAL and MPO concentrations (OR 1.72, 95% CI 1.06–2.85, *p‐*value = 0.03) (Table 4).

**Table 4:** Predictors of calprotectin < 25th percentile and myeloperoxidase < 25th percentile: multivariable models.

Predictor variable	Outcome											
	Calprotectin < 25th Percentile		Calprotectin < 25th percentile		Myeloperoxidase < 25th percentile		Myeloperoxidase < 25th percentile		Myeloperoxidase & calprotectin < 25th percentile^a^		Myeloperoxidase & calprotectin < 25th percentile^a^	
	Minimally‐adjusted model^a^		Multivariable model^b^		Minimally‐adjusted model^a^		Multivariable model^b^		Minimally‐adjusted model^a^		Multivariable model^b^	
	OR (95% CI)	*p*‐value	OR (95% CI)	*p*‐value	OR (95% CI)	*p*‐value	OR (95% CI)	*p*‐value	OR (95% CI)	*p*‐value	OR (95% CI)	*p*‐value
Biochemical variables												
Hemoglobin (g/dL)					1.39 (0.96, 2.02)	0.08	1.28 (0.87, 1.90)	0.27				
RBP (umol/L)^c^					5.10 (1.30, 19.90)	0.02	6.72 (1.67, 29.68)	0.01	4.03 (0.91, 17.87)	0.07	3.94 (0.88, 18.70)	0.08
AGP g/L					0.50 (0.25, 1.02)	0.06	0.37 (0.16, 0.82)	0.02				
Anthropometric variables												
MUAC‐for‐age Z‐score	1.60 (1.04, 2.47)	0.03	1.60 (1.04, 2.47)	0.03					1.72 (1.05, 2.80)	0.03	1.71 (1.05, 2.86)	0.03

Abbreviations: AGP, alpha‐1‐acid glycoprotein; BMI, body mass index; CI, confidence interval; MUAC, middle‐upper arm circumference; OR, odds ratio; RBP, retinol‐binding protein.

^a^ Logistic regression models are minimally adjusted for child age.

^b^ Multivariable logistic regression models are adjusted for child age, and all variables associated.

^c^ RBP concentration was adjusted for CRP and AGP using the BRINDA regression correction approach.

## 4 Discussion

In this study of EED biomarkers among young Bangladeshi children, we observed that over 90% and 70% of children exhibited elevated concentrations of CAL (> 160 ug/g) and MPO (> 2000 ng/mL), respectively. We identified several anthropometric indicators and micronutrient biomarkers that were associated with lower concentrations of CAL and MPO, indicating protective associations. In multivariable models, children with higher MUACZ were more likely to have lower CAL concentrations. Increased RBP concentrations were associated with increased odds of lower MPO, whereas children with higher AGP concentrations were less likely to have lower MPO concentrations. Children with higher MUACZ were also more likely to have both CAL and MPO in the lowest quartile.

We observed a markedly high prevalence of elevated EED markers among young children in this study. We note the challenges in measuring these markers in pediatric populations, especially CAL, as breastfeeding, age and laboratory methods can all influence concentrations. Some studies have attempted to characterize reference values in healthy pediatric populations using similar ELISA methods; for example, median CAL concentrations of 96.1 µg/g (5th, 95th percentile 10–398) have been reported among healthy Chinese children 9–12 months of age (Li 2015), and 40.6 µg/g (IQR 14.2–100.3) among healthy Hungarian children 4–12 months of age (Kiss 2025). Fewer studies have established healthy reference ranges for MPO in children; however, a cutoff of < 2000 ng/g is frequently used to distinguish normal from elevated levels in pediatric populations. In this context, the biomarker concentrations observed in the present study suggest markedly elevated intestinal inflammation among young children in this population.

Given a lack of standardized diagnostic criteria for EED and the fact that most children in our sample exceeded the ELISA‐defined cutoffs for elevated biomarker values, we employed a positive deviance approach based on the observed distribution of EED biomarkers within this population. Similar approaches have been used in other studies of EED biomarkers in Bangladesh (Arndt 2016). Prior research conducted among children of a similar age has also reported an elevated prevalence of EED markers in this area of Dhaka, Bangladesh (Hossain 2023; Fahim 2018; Arndt 2016). Notably, a recent study of 110 children residing in the *Bauniabadh* slum in Mirpur, Dhaka identified chronic, non‐specific duodenitis characterized by villus atrophy, crypt hyperplasia, and lymphatic infiltration in over 90% of children 12–18 months using intestinal biopsy, which is considered the gold‐standard measurement for diagnosis of EED (Hossain 2023). This evidence is consistent with the results from our study and emphasizes the urgent need to address EED among young children in this region.

We found that MUACZ and WLZ were associated with lower CAL concentrations in minimally‐adjusted models. CAL is elevated in the presence of inflammation of the intestinal mucosa (Jukic 2021; Stríz and Trebichavský 2004; Vaos 2013; Pathirana 2018). Several studies have observed associations between CAL and anthropometric deficits in young children in Bangladesh (Arndt 2016), and other lower‐middle income country contexts in South America, Asia and Africa (Syed et al. 2016; Kosek 2017). Although anthropometric indicators were not associated with lower MPO concentrations in our study population, several biomarkers of micronutrient and inflammatory status were associated with MPO. MPO induces significant immune activation and oxidative stress in the colon (Chami 2018). Vitamin A also plays a major role in immune function, as well as maintaining epithelial integrity in the intestine (McCullough et al. 1999). Indeed, children with higher RBP concentrations in this study were more likely to have MPO concentrations in the lowest quartile. Several other studies have also noted an inverse association between MPO and RBP levels (Fahim 2022; Liu 2014). We note that routine high‐dose vitamin A supplementation was provided in the study area, which likely influenced RBP concentrations. Accordingly, these findings should be interpreted with caution. Even so, the observed association remains informative and suggests a potential relationship between EED markers and RBP concentrations.

AGP, a marker of systemic inflammation, was associated with a reduced odds of lower MPO (i.e. higher MPO concentrations). Although AGP is considered a positive acute‐phase protein (Luo 2015), it rises more slowly and remains elevated for a longer period than CRP, reflecting a more chronic inflammatory response (Gannon 2019). Indeed, persistent immune activation was apparent in these children, reflected by a greater proportion of children with elevated AGP (22%) versus CRP (8%). Our findings suggest that children who experienced lower levels of systemic inflammation also had lower enteric inflammation. This aligns with a proposed mechanism of EED, where microbial translocation from the gut into systemic circulation triggers systemic inflammation (Syed et al. 2016). Other studies have also linked MPO to AGP concentrations (Kosek 2017).

In multivariable models, MUACZ was significantly associated with greater odds of lower CAL and MPO or better gut health (OR 1.71, 95% CI 1.05–2.86). This finding may reflect a more acute response to EED, whereby intestinal inflammation initially manifests as nutrient malabsorbtion and deficits in ponderal growth, with subsequent impacts on linear growth later in life. Indeed, some research, including other studies in Bangladesh, has reported associations between EED and weight gain, but not linear growth in children under 2 years of age (Kosek 2017; Arndt 2016; Campbell 2018). We note that we did not observe a significant association between LAZ and EED biomarkers, although many previous studies have identified an association between LAZ and EED (Kosek 2013; Weisz 2012; Lunn et al. 1991; Goto et al. 2009). However, evidence suggests that growth faltering does not follow a simple sequence of early WAZ decline followed by subsequent LAZ decline. Rather, linear growth may be influenced by additional factors not measured here, including prenatal factors and birth length, with birth length consistently identified as one of the strongest predictors of length attained at 2 years (Krebs 2022).

These findings emphasize the need for interventions that aim to reduce EED. Such high prevalence and severity of EED may seriously hinder the effectiveness of nutritional interventions. Several studies have demonstrated limited benefits from micronutrient supplementation and complementary feeding interventions in improving child growth in low‐ and middle‐income countries where EED is common, including Bangladesh (Victora 2021; Tickell et al. 2019b; Imdad 2023; Suchdev 2020; Tam 2020; Millward 2017). Indeed, in the main ZiPT trial, daily zinc supplementation was not enough to improve linear growth in this population (Islam 2022). The link between EED biomarkers and growth reinforces that multisectoral interventions aimed at sustainably reducing and preventing EED, including water, sanitation and hygiene (WASH) interventions to reduce enteropathogen exposure, vaccination, probiotic interventions, maternal health and poverty reduction, may be important to prioritize alongside nutritional interventions (Syed et al. 2016; Tickell et al. 2019b; Mbuya and Humphrey 2016).

Our study has key strengths, including the assessment of biomarkers of micronutrient status and systemic inflammation, which allowed us to explore potential mechanistic insights between EED markers and nutritional status. This study also has several limitations. Because most children exhibited elevated biomarker levels, we relied on a percentile‐based approach that defined the reference group as the lowest 25th percentile, which may limit the generalizability of our findings to other settings. Additionally, the relatively small size of this reference group may have reduced our statistical power to detect meaningful associations. Further, our findings are based on cross‐sectional data, which limits our ability to establish causal relationships or infer temporal dynamics between risk factor variables and EED biomarkers. Building on this, because acute infections may transiently elevate inflammatory biomarkers, we cannot exclude the possibility that recent morbidity influenced biomarker concentrations. However, given the high prevalence of EED and the limited number of asymptomatic children, we believe observed associations with EED markers remain informative, though they should be interpreted with caution. Lastly, we were limited to only two biomarkers of EED: CAL and MPO. Intestinal biopsy remains the gold standard test for diagnosing EED (Denno et al. 2017), however, this procedure is highly invasive and was not possible to perform in a field setting. While other biomarkers of EED have been identified, such as fecal neopterin and fecal alpha‐1 antitrypsin (Keusch 2014), CAL and MPO are widely used to identify EED, ensuring comparability with existing research in this field.

In conclusion, our study identified important associations between anthropometric variables, biomarkers of micronutrient and inflammatory status, and EED biomarkers among young children in Dhaka, Bangladesh. Specifically, MUACZ, RBP and AGP were associated with biomarkers of EED. These findings highlight the intricate relationship between EED biomarkers, immune function, and child growth, and suggest that interventions aimed at reducing the burden of EED may help to enhance the effectiveness of interventions among nutritionally vulnerable children to sustainably improve health and growth outcomes.

## Author Contributions

L.T., J.M.L., J.L.E.W., A.M.K., R.A.S., M.M.I., R.E.B., N.F.K. and C.M.M. designed and conducted the study. L.T. and C.A. performed the data analysis. L.T. wrote the initial draft of the manuscript. All authors contributed to manuscript revisions. All authors read and approved the final manuscript.

## Conflicts of Interest

The authors declare no conflicts of interest.

## Data Availability

The data that support the findings of this study are available on request from the corresponding author. The data are not publicly available due to privacy or ethical restrictions.
